# The Association Between Classroom Quality and the Social Competence of Autistic Preschool-Age Boys

**DOI:** 10.1007/s10803-025-06747-6

**Published:** 2025-02-11

**Authors:** David Oppenheim, Smadar Dolev, Lior Hamburger, Rony Lottan, Shimrit Kunst, Jenny Friedelman, Michal Mottes-Peleg, Nurit Yirmiya

**Affiliations:** 1 School of Psychological Sciences and Center for the Study of Child Development, University of Haifa, Haifa, Israel; 2 Department of Early Childhood Education, Oranim Academic College of Education, Kiryat Tiv’on, Israel; 3 Department of Psychology, The Hebrew University of Jerusalem, Jerusalem, Israel

**Keywords:** Autism, Classroom quality, Social competence, Special education

## Abstract

Research on the impact of the classroom environment on neurotypical children has demonstrated that higher classroom quality contributes to children’s development, but whether this is also true with regard to autistic preschoolers has not been examined. Therefore, the goal of this study was to address this gap hypothesizing that higher classroom quality would be associated with higher child social competence both in and outside the classroom. The quality of the classrooms of 43 autistic preschooler boys was assessed by observation, and children’s social competence in preschool was assessed by observation and teacher-report, and outside preschool by observing children’s interactions with an unfamiliar adult. Controlling for the severity of the boys’ symptoms, results revealed that higher classroom emotional support and organization was associated with higher child social competence as observed and reported by teachers in preschool, and with the boys’ involvement with of an unfamiliar adult during play. The quality of the classroom environment was associated with the social skills of autistic boys.

## Introduction

Research on the impact of the classroom environment on the development of neurotypical children has shown that higher classroom quality contributes to children’s development, including their self-regulation, academic skills, and, particularly relevant for the present study, their social competence (Hamre, 2014). Whether this is also true with regard to special education preschools, and specifically with regard to autistic preschoolers has not been examined. This is a particularly important question because autistic preschoolers experience significant social difficulties, and can benefit from supportive environments that are attuned to their individual characteristics and needs, perhaps even more than typically developing young children (Baker et al., 2010; Haven et al., 2014). Therefore, the goal of this study was to examine whether, with regard to preschool-age autistic boys, the quality of the classroom environment would be associated with the boys’ social competence both in preschool and outside preschool.

Typically developing children utilize a wide range of social skills when interacting with peers, whereas autistic children have significant challenges in the development and utilization of such skills (Charlop et al., 2010). Although perhaps not less motivated to connect with their peers (Jaswal & Akhtar, 2019), they may have difficulties using the appropriate social skills. Autistic children show less interest in interacting with others, engage in more solitary play, are less preferred as playmates, experience fewer reciprocal friendships, and have lower quality interactions (Baker et al., 2007; Charlop et al., 2010; Cotugno, 2009; Kasari et al., 2011; McConnell, 2002). However, alongside these challenges there are also considerable individual differences in autistic children’s social skills, and one contributor to these differences is their prior social experiences. For example, examining the contribution of children’s interactions with their parents to their social skills, Haven et al. (2014) found that cohesion and warmth in parent–child interactions were related to children’s social skills for both Typically Developing (TD) and autistic children, and Meek et al., (2012) found that child-initiated joint engagement with parents was positively related to social competence and less exclusion by peers. To the best of our knowledge this is the first study to examine the contribution of children’s experiences outside the family, in preschool, to their social skills.

Autistic preschoolers, like their neurotypical peers, spend a large portion of their time in preschools with peers and educational staff (although this may vary widely depending on educational policies and opportunities, but was the case regarding the preschoolers in this study). As mentioned above, in the case of neurotypical children, high quality early childhood settings have been shown to have a positive impact on children’s development. For example, higher-quality child care predicted better preacademic skills and language performance at 4.5 years even after controlling for two other well-recognized influences on early development: parenting and poverty (NICHD ECCRN 2002). Following the same sample to the age of 15 years, higher quality early care predicted higher cognitive–academic achievement and also youth reports of less externalizing behavior (Vandell et al., 2010).

Focusing more specifically on teacher–child interactions, Hamre and Pianta (2007) described three domains that promote positive development: Emotional support (sensitivity to the children, positive relationships, and respect to the child’s point of view), Classroom organization (routines that keep children engaged in meaningful and interesting activities, proactive management of child behavior), and Instructional support (Connecting learning to children’s lives, feedback that expands children’s understanding, frequent conversations). Evidence supports the importance of these domains (Hamre, 2014). For example, pertaining to the Emotional Support domain, children with sensitive teachers with whom they develop positive relationships develop more optimal prosocial (Johnson et al., 2013) and self-regulatory (Williford et al., 2013) skills compared to children whose teachers are less sensitive, and children with teachers who offer warm supportive care show less of the expected increase in stress over the day (Hatfield et al., 2013) compared with children whose teachers offer less supportive care. Beyond these correlational findings, intervention studies showed that proactive management of children’s attention by teachers, pertaining to the Classroom Organization domain, was associated with less time off task and with better behavioral control (Rimm-Kaufman et al., 2014). Furthermore, teacher training that targeted teacher–child interactions improved teachers’ practices, with the children benefiting as indicated by better academic, social, and self-regulatory functioning (e.g., Mashburn et al., 2010; Raver et al., 2011).

Thus, there is considerable evidence that neurotypical children benefit from high quality classroom environments. To the best of our knowledge, however, there is little if any similar evidence in the case of autistic children. This lacuna is significant for at least two reasons: First, there is a consensus regarding the importance of intervening early and intensively with young autistic children, and that such intervention should not be circumscribed to therapy sessions but should envelop the child throughout the day (Schreibman et al., 2015). For many autistic children the preschool setting is the most significant social context outside the family, and as such has a unique potential to foster their independence and competence (Siller et al., 2024). Second, Haven et al. (2014) and Baker et al. (2010) argued that autistic children may be impacted by variations in the quality of the environment even more than neurotypical children. This idea was raised regarding parent–child interactions, but can be equally true regarding teacher–child interactions and the classroom environment more generally.

### The Present Study

The goal of the study was to examine the associations between the quality of the preschool environment of young autistic preschool age boys and the children’s social competence. Specifically, teacher–child interactions were assessed using the Classroom Assessment Scoring System (CLASS; Pianta, 2008). The CLASS has been extensively employed in studies of mainstream education (Hamre, 2014) but has yet to be employed in special education preschools. It was chosen nonetheless because it focuses on teacher–child interactions and the overall emotional climate of the classroom that are thought to be as important for autistic children as for neurotypical children (Siller et al., 2024). The boys’ social competence was assessed in preschool by observation and teacher report, and outside preschool by observing the boys’ responsiveness and involvement with an unfamiliar adult during play. The hypothesis was that higher preschool quality would be associated with higher social competence in preschool and with higher levels of responsiveness and involvement with an unfamiliar adult outside of the preschool. Because the boys’ social competence and interactions with an examiner could be impacted by the severity of their autism symptoms, symptom severity as assessed by the Autism Diagnostic Observation Schedule (ADOS; Lord et al., 2000) was statistically controlled.

Only boys were included in the study. This was done because it has been argued that autism may present differently in boys and girls (Hull et al., 2020). Therefore, particularly in a study of social behavior, studying autistic boys and girls as one group may conceal gender differences. Ideally separate and large groups of boys and girls should be studied, but we chose to focus on boys because autism is approximately 4 times more prevalent in boys compared to girls (In the US: Maenner et al., 2020; In Israel: Davidovitch et al., 2013). Additionally, and strictly from a statistical power standpoint, combining boys and girls would decrease the size of each group (considering a given sample size) which would decrease the study’s power.

## Method

### Participants

Forty-three autistic preschool age boys, a sub-sample of a larger study of autistic preschoolers and their families (Oppenheim et al., 2023) participated. The boys’ ages ranged between 29 and 67 months (*M* = 51.37, *SD* = 10.05). Fifty-two percent of the boys were first born and 74% had siblings. Almost all of the boys (*n* = 42, 98%) were in special education classes that included only autistic children. Each of the boys was in a different class with a different teacher. The boys spent a mean of 41.58 h per week in preschool (*SD* = 11.88). Information regarding teacher age, teacher education, number of children in the class, and ratio of staff to children was obtained because these factors might impact classroom quality, and number of months each of the boys was with the teacher with whom he was observed was also recorded (see Table 3). The boys’ families were recruited through treatment centers and through social media. Inclusion criteria included being male, a known diagnosis of ASD for the child which was confirmed as part of the study, age between 2.5 and 6 years, and no known additional medical diagnoses. Parents were given purchase coupons for their participation as well as a summary of their son’s developmental assessment and of the observation at preschool.

### Procedure

The study included two laboratory visits and three preschool observations. The first laboratory visit included the Autism Diagnostic Observation Schedule (ADOS; Lord et al., 2000). The second laboratory visit, conducted 1–2 weeks later, included an observation of the boys playing with an unfamiliar examiner to assess their social behavior. Both visits included additional assessments that were not part of this study.

Following the laboratory visits, three preschool observations were conducted. Importantly, the preschool observations were conducted only after the children had adjusted to preschool. Each of the first two visits included a 2-h observation to assess children’s social skills using the Social Skills Q-sort (SSQ; Locke et al., 2014). The visits were conducted two weeks apart, and in the second visit the preschool teacher also completed the SSQ. In the third visit different observers completed the CLASS (Pianta, 2008) to assess the quality of the preschool environment. Teachers were given a shopping coupon as a token of appreciation.

### Measures

#### Autism Assessment

*Autism Diagnostic Observation Schedule* (ADOS; Lord et al., 2000). The ADOS is a standardized and widely used assessment for diagnosing individuals with ASD through interactions with a trained professional. It consists of 4 modules of which one is administered based on the child’s level of expressive language. Eighteen children were assessed using module 1, 21 using module 2, and 4 using module 3. The ADOS diagnostic algorithm was used (Gotham et al., 2007), and combined with the child’s age a Calibrated Severity Score (CSS; *M* = 7.01, *SD* = 1.58, *range* = 4–10) was calculated (Gotham et al., 2009). The blind assessors’ reliability for 17 children (21% of the full sample) was maintained on an ongoing basis and mean percent agreement was 87% (range: 78 to 94%).

*Social Communication Questionnaire* (SCQ; Rutter et al., 2003). The SCQ is a 40-item autism symptom checklist that parents score as present (1) or absent (0). The points are summed and the cut-off for ASD for young children is ≥ 11 (Wiggins et al., 2019).

The children’s ASD diagnoses were confirmed based on the ADOS and the SCQ as well as the clinical judgement of an experienced licensed psychologist. All community diagnoses were confirmed.

#### Assessment of Classroom Quality

Classroom quality was assessed using the Classroom Assessment Scoring System (CLASS K-3; Pianta, 2008) which is widely used for assessing classroom quality. Observers completed the CLASS after observing three separate 20-min episodes. The episodes were selected to include various classroom activities including structured (e.g., circle time) and unstructured (i.e. free play) activities. The CLASS assesses three domains: Emotional support, classroom organization, and learning support, of which the first two were used in this study (learning support was not used because learning processes in the autism preschools observed in this study are markedly different from those in mainstream preschools, for which the CLASS was developed). Each domain includes several scales (described below) rated using a 7-point scale ranging from “1” (classroom is low on that dimension) to “7” (classroom is high on that dimension).

The *emotional support* domain consists of four scales: positive climate (the extent to which the teacher and children display enthusiasm, enjoyment, and respect during classroom interactions); negative climate (the degree to which the teacher and children present a negative emotional and social tone); teacher sensitivity (the extent to which the teacher provides comfort, reassurance, and encouragement); regard for children’s perspective (the extent to which activities chosen by the teacher emphasize children’s interests and encourage student autonomy).

The *classroom organization* domain consists of three scales: behavior management (the extent to which the teacher uses effective methods to prevent and redirect children’s misbehaviors); productivity (the extent to which the teacher manages instructional time and routines so that children learn and make progress); instructional learning formats (the teacher’s use of available activities, methods of presentation, use of groupings, and range of materials to maximize children’s engagement).

Both coders were trained to reliability by a master coder trained by *Teachstone* prior to the study. Additionally, 24% (*n* = 32) of the observations were conducted by both coders to assess inter-rater reliability which was 90.1% (range 71.8–100%).

Following the CLASS manual (Pianta, 2008) and as commonly done (e.g., Dolev et al., 2021) the mean of the four Emotional Support scales (with negative climate reversed) was computed to form an Emotional Support composite score, and similarly, the mean of the three Classroom Organization dimensions was computed to form a Classroom Organization composite score. The Emotional Support and Classroom Organization composite scores were strongly associated (*r* = .76, *p* < .001) and therefore the mean of the two scores was computed to form a Classroom Quality composite score.

#### Assessment of Boys’ Social Competence in Preschool

The boys’ social competence at preschool was assessed using the *Social Skills Q-Sort* (SSQ; Locke et al., 2014). The SSQ assesses the social skills of school-age autistic children in their educational setting and includes 100 items which teachers sort into nine piles of 11 items each (middle pile includes 12 items), ranging from least (coded 1) to most (coded 9) characteristic of the child. The SSQ items use general descriptions that can apply to preschoolers as well, but we added to each item descriptions to clarify how the general description applies to preschoolers’ behavior. For example, the following descriptors were added to the item “Elicits attention negatively”: High score: The child may grab a toy from another child or ignore the teacher’s prohibition waiting to see the response; Low score: The child elicits attention positively. For example, complies with the teacher’s request and anticipates recognition; shares a toy with a peer and waits for a positive response. Each child’s sort is correlated with a sort of a prototypic socially competent TD child provided by the SSQ developers to yield a teacher-reported Social Competence score. The resulting correlations were normalized using Fisher’s *r* to *Z* formula.

An 81-item observer SSQ was developed for the present study omitting from the teacher-reported SSQ items (available from author) that require long-term familiarity with the child. Observers blinded to any information about the child or the family completed the SSQ as soon as possible following a 2-h observation in the boys’ preschools that included both structured and unstructured activities. This involved sorting the cards into 9 piles of 9 cards each, ranging from least- (coded 1) to most-characteristic (coded 9) of the child. Two preschool observations of each boy were conducted 1 week apart by the same observer yielding two sorts for each child. As in the teacher SSQ, observer SSQ scores were computed by correlating each boy’s sort with the sort of the prototypic socially competent TD child (Locke et al., 2014) and normalizing the correlation using Fisher’s *r* to *Z* formula. The correlation between the SSQ scores from the first and second observations was very high (*r* = .88, *p* < .001) and therefore a mean observer SSQ score was computed. Inter-rater reliability was calculated for 20% of the observations and was ICC = .70.

#### Assessment of the Boys’ Interactions with the Examiner

The boys were observed playing with an unfamiliar examiner for 5 min (completing a puzzle at a level of difficulty appropriate for the child). The boys’ behavior was coded using the following two 1–7 scales which were adapted from Biringen’s Emotional Availability Scales (EAS; 4th edition; Biringen et al., 2014): *Child Responsiveness to the Adult* which assesses the child’s willingness to follow the adult’s suggestions and the display of pleasure in interacting. The overall score is derived taking into consideration the following sub-scales: positivity of affect and regulation of emotions and behavior; appropriate responsiveness to adult initiations; age-appropriate exploration and autonomy; lack of active avoidance; and lack of excessive task-oriented attitude. *Child Involvement with the Adult* which assesses the degree to which the child attends to and engages the adult in play. The overall score is derived taking into consideration the following sub-scales: Use of brief, one-time, simple initiatives; the use of elaborative initiatives that lead to an extended period of engagement; modulated use of eye contact and looking for engagement with the adult; positive body positioning; and the use of verbal involvement. The adaptation of the original Responsiveness and Involvement EAS scales for the present study involved removing two sub-scales from each of the original two scales (Positive physical contact and Lack of role reversal for the Responsiveness scale, and Use of the adult and Lack of over involvement for the involvement scale) because they were deemed not pertinent to children’s interactions with an unfamiliar adult (as opposed to a parent).

The coders were blinded to all information about the families. Inter-rater reliability was established between each of the coders and a master coder who was trained to reliability on the EAS by the scales’ developer (Biringen). Inter-rater reliabilities were assessed for 36.14% of the observations and were ICC = .87 for Child Responsiveness and ICC = .82 for Child Involvement.

#### Analytic Plan

Descriptive statistics and associations between the study variables (CLASS Emotional Support and Classroom Organization scores, SSQ scores reported by observer and teacher, and the boys’ Responsiveness and Involvement with an unfamiliar examiner scores) and demographic/background variables are reported first. Intercorrelations among the study variables are reported subsequently. Finally, four hierarchical regressions were conducted predicting the two SSQ scores and boys’ Responsiveness and Involvement scores. The boys’ CSS scores and age and teacher age were entered in the first step as control variables, and the Classroom Quality scores was entered in the second step.

## Results

### Preliminary Analyses

The descriptive statistics of the study variables are presented in Table 1 and the associations between the study variables and demographic/background and teacher variables are presented in Table 2. The Classroom Quality score was unrelated to any of the child, teacher, and classroom variables. The observer SSQ score was negatively associated with the boys’ CSS and with teachers’ age. The teacher-reported SSQ was also negatively associated with the boys’ CSS. Finally, children’s Responsiveness and Involvement with the examiner were positively associated with the boys’ age and negatively associated with CSS. Consequently, the boys’ age and CSS and teachers’ age were controlled in subsequent analyses. The correlations between the study variables are presented in Table 3.

**Table 1:** Descriptive statistics of the study variables

Variable	*M*	*SD*	Min–max
CLASS – Quality	4.80	.76	2.81–5.83
SSQ – Observer	0.39	.43	− .45–1.09
SSQ – Teacher	.10	.37	− .76–.89
Responsiveness with examiner	4.73	1.41	1.00–7.00
Involvement with examiner	3.53	1.65	1.00–7.00

*CLASS* Classroom Assessment Scoring System; *SSQ* Social Skills Q-Sort

**Table 2:** Descriptive statistics and correlations between study variables and background/control variables

	Child age (months)		CSS		Teacher age (years)		Teacher education		Number of children in class		Ratio of staff to children		Months child is with teacher	
	*M*	*SD*	*M*	*SD*	*M*	*SD*	*B.A.*	*M.A.*	*M*	*SD*	*M*	*SD*	*M*	*SD*
	51.37	10.05	6.81	1.37	32.57	8.18	83.3%	16.6%	8.38	1.30	.36	.14	16.33	10.04
CLASS quality	.09		− .06		− .12		− .03		− .18		.08		.11	
Observer SSQ	.09		− .27*		− .26*		− .01		− .14		.14		.03	
Teacher SSQ	− .07		− .33**		− .17		− .05		− .06		.10		− .08	
Responsiveness with examiner	.29*		− .43**		− .10		.05		− .02		.00		.17	
Involvement with examiner	.28*		− .23^+^		− .15		.17		− .10		.01		.21	

*CLASS* Classroom Assessment Scoring System; *SSQ* Social Skills Q-sort; *B.A.* Bachelor of Arts; *M.A.* Master of Arts

^+^*p* < .10 *p < .05, ** *p* < .01, one-tailed

**Table 3:** Intercorrelations of the study variables

	1	2	3	4	5
1. CLASS quality	1.00	.39**	.34*	.29*	.46**
2. Observer SSQ		1.00	.75***	.45**	.58***
3. Teacher SSQ			1.00	.37**	.38**
4. Responsiveness with examiner				1.00	.73***
5. Involvement with examiner					1.00

*CLASS* Classroom Assessment Scoring System; *SSQ* Social Skills Q-sort

*p < .05, **p < .01, ***p < .001, one-tailed

#### Predicting Children’s Social Skills

The associations between classroom quality and the boys’ social skills in preschool controlling for the boys’ CSS and age and teachers’ age were examined by two hierarchical regression equations with children’s observer-reported SSQ and teacher-reported SSQ scores as the dependent variables in each one of the regressions respectively (see Table 4).

**Table 4:** Stepwise regressions predicting children’s social skills from classroom quality (final regression)

Predictors	Observer SSQ			Teacher SSQ			Responsiveness with examiner			Involvement with examiner		
	$B$	*SE B*	$\beta$	$B$	*SE B*	$\beta$	$B$	*SE B*	$\beta$	$B$	*SE B*	$\beta$
Step 1												
Child age	.00	.00	.00	.00	.00	− .18	.02	.02	.17	.03	.02	.18
Child CSS	− .06	.04	− .19	− .09	.04	− .33**	− .37	.15	− .36*	− .14	.17	− .12
Teacher age	− .01	.00	− .18	.00	.00	− .08	.00	.02	− .03	− .01	.02	− .08
Step 2												
Classroom quality	.21	.08	.39**	.16	.07	.32*	.48	.26	.26^+^	.98	.29	.46**
Final regression	*F*_(4, 37)_ = 3.32, *p* < .05			*F*_(4, 37)_ = 3.12, *p* < .001			*F*_(4, 37)_ = 3.71, *p* < .05			*F*_(4, 37)_ = 4.35, *p* < .001		

*SSQ* Social Skills Q-sort; *CSS* Calibrated Severity Score

^+^*p* < .10, *p < 0.05, **p < 0.01

In predicting observer SSQ scores, the boys’ CSS scores and age and teachers’ age were entered first and accounted for 11.7% of the variance (*F*_(3, 38)_ = 1.68, *ns*) and Classroom Quality was entered second and accounted for an additional 14.7% of the variance (*F*_(1, 37)_ = 7.39, *p* < .01).

In predicting the teacher SSQ scores, the boys’ CSS scores and age and teachers’ age were entered first and accounted for 14.9% of the variance (*F*_(3, 38)_ = 2.22, *ns*), and Classroom Quality was entered second and accounted for an additional 10.3% of the variance (*F*_(1, 37)_ = 5.11, *p* < .05).

To examine the associations between classroom quality and the boys’ Responsiveness and Involvement when interacting with an examiner controlling for the boys’ CSS and age and teacher age, two additional hierarchical regression equations were conducted with the boys’ Responsiveness and Involvement scores as the dependent variables in each one of the regressions respectively (see Table 4).

In predicting the boys’ Responsiveness with the examiner, the boys’ CSS scores and age and teacher age were entered first and accounted for 22.1% of the variance (*F*_(3, 38)_ = 3.59, *p* < .05) and Classroom Quality was entered second and accounted for an additional 6.5% of the variance (*F*_(1, 37)_ = 3.39, *p* < .10).

Finally, In predicting the boys’ Involvement with the examiner, the boys’ CSS scores and age and teachers’ age were entered first and accounted for 11.6% of the variance (*F*_(3, 38)_ = 1.65, *ns*) and Classroom Quality was entered second and accounted for an additional 20.5% of the variance (*F*_(1, 37)_ = 11.14, *p* < .01).

## Discussion

The goal of the study was to examine the hypothesis that higher quality of the preschool environment will be associated with higher levels of social skills of autistic boys both in the preschool context and outside this context, when interacting with an unfamiliar adult. Supporting this hypothesis, a composite score based on the emotional support and organization CLASS dimensions was associated with the boys’ social competence as assessed by observation at preschool using the SSQ, and as reported by the preschool teachers who also completed the SSQ. In addition, the CLASS quality score was associated with the boys’ interactions with an unfamiliar examiner, particularly their involvement of the examiner in their play, and also, albeit only with marginal statistical significance, with their responsiveness to the examiner. Importantly, the severity of the boys’ symptoms, which was also associated with their social competence (as expected) was controlled in these analyses.

What is it about high quality classrooms, those characterized by relatively high emotional support and classroom organization, the might support social competence in autistic preschoolers? Difficulties in social behavior and communication in the peer-group context are common among autistic children, as reviewed earlier. However, the preschool setting is not only the context in which these difficulties manifest, but also a useful setting for addressing these difficulties and fostering development and growth. This is because children spend many hours in preschool, are surrounded by many social partners including adults and children with whom they often wish to interact (Jaswal & Akhtar, 2019), and need to deal with many and diverse social interactions. In fact, promoting children’s competencies, including their social competence, is one of the declared goals of special education preschools for autistic children in Israel as defined by the Ministry of Education in Israel. Additionally, while adults, and particularly preschool teachers, tend to support children by adjusting their behavior to the child’s difficulties, children’s peers are less likely to be as accommodating as the adults (Clifford et al., 2010), thus providing an opportunity for the adults to mediate peer interactions. Emotionally supportive classroom environments characterized in the CLASS by high levels of positive interaction, low levels of negative interactions, sensitivity to the child’s signals, and regard for the child’s point of view (Hamre, 2014), are likely to provide the needed mediation by understanding children’s needs and difficulties at a given moment and supporting children’s efforts to interact with others.

The classroom quality composite was also included the CLASS organization dimension. Classrooms that score high on this dimension are characterized by clear rules and a well-structured flow of the day, a variety of interesting and stimulating activities, and teachers who intervene to prevent escalations and set appropriate limits. These are likely to support the development of children’s independent capacity to control and regulate their emotions and behavior (Hamre, 2014; Williford et al., 2013). These conditions also allow children to operate within the zone of proximal development and take on challenges in various domains, including the social domain.

Support for the possible impact of the classroom environment and the boys’ social competence was also provided by the links between classroom quality and the boys’ behavior *outside* the classroom, interacting with an unfamiliar examiner. Boys who experienced higher quality classrooms involved the examiner more when interacting with her and were more likely to initiate interactions with her. Additionally, they were more responsive to the initiations of the examiner (although the latter finding was only marginally significant when the boys’ severity of symptoms was controlled). The findings regarding responsiveness to- and involvement of the examiner are significant because both behaviors are significant challenges for many autistic children (Mundy et al., 1986). Perhaps the sensitivity and support coupled with expectations for self-regulation characterizing high quality classrooms promoted the boys’ interactions with an unfamiliar yet friendly adult. Although the preschool and lab contexts are different on many dimensions, particularly because the lab context did not include peers, in both cases the children interact with an adult outside the family. This may have fostered the application of skills acquired in the classroom to the interactions with an examiner.

When considering the associations between the CLASS quality score and the SSQ it is possible to argue that the association between the two is because CLASS observers are exposed to the behavior of the child involved in the study, and similarly the SSQ observers are exposed to the classroom climate. This cannot be ruled out: Although the two observations were conducted by different observers on different days, the overlap between them is inevitable. It is not possible to assess the class quality without the children, or the target child’s social competence in preschool outside the classroom. Nonetheless, it is important to note that the focus of the CLASS is on the entire classroom, with a primary focus on the teacher and other adults, and the focus of the SSQ is on the behavior of a single target child.

A related issue involves direction of effects: Because both the CLASS and SSQ were assessed concurrently it is possible to argue that the link between the two is not a function of the impact of the classroom quality on the child’s behavior but the reverse. This cannot be ruled out but is less likely, because any single boy involved in the study was only one of many children in the classroom (approximately eight on average, none of whom were involved in the study other than the target child). It is less plausible that a single boys’ behavior will impact the teachers’ interactions with all children. The lack of association between each target boy’s symptom severity (i.e., CSS score) and the CLASS score supports this argument.

It is also possible to argue that the link between class quality and children’s social competence is due to differential placement, in which children are placed in different preschools depending on characteristics such as severity of symptoms. This may be plausible although more severe symptoms need not necessarily lead to poorer quality of the preschool. These arguments notwithstanding, in order to support causal statements regarding the impact of classroom quality on children’s social competence intervention studies using an RCT design are needed.

Two methodological strengths bolster the results of the study. First, the study’s main three constructs, classroom quality, children’s social competence in preschool, and their interactions with an examiner, were assessed by independent observations. Second, the children’s social competence in preschool was assessed by observation (as mentioned) but also by teacher-report. Assessments of children’s social competence using trained observers in preschool have the advantage of the standardization obtained using observer training and inter-rater reliability. Teacher reports, in contrast, may lack in standardization because teachers were not trained on the SSQ, but have the advantage of extensive and deep familiarity with the child over time. The consistency of the findings involving both measures, and the association between them, increase the confidence in the study results.

Two limitations are also noteworthy. First, the study involved only boys, and because, as mentioned earlier, the social difficulties of autistic girls may manifest differently (Gould, 2017; Hull et al., 2020), additional research with girls is needed. Second, the boys in this study were all enrolled in special education preschools which are relatively small and composed only of children with an ASD diagnosis. Whether similar findings apply to children in different educational systems such as those that are inclusive, have different class size, and in which teachers are trained differently, remains an open question. Nonetheless, the findings highlight the potential importance of the quality of the preschool environment, particularly the emotional support it provides and the level of class organization, for supporting the social development of autistic preschoolers.

The findings have important implications for educational settings. Creating a positive climate, responding sensitively to children’s behaviors, and seeing their interests as significant appears to promote the social skills of autistic children. Additionally, managing behaviors of children with ASD, while challenging, is essential for promoting their social engagement, and can be achieved through respectful, positive, and productive approaches, alongside creating engaging and varied activities. Interventions that target these dimensions and are aimed at improving the classroom climate for typically developing children, such as MyTeachingPartner (MTP) (Allen et al., 2011; Pianta et al., 2002) have shown improvement in teacher-student interactions in preschool settings (Hamre et al., 2012; Pianta et al., 2008; Tolan et al., 2020), and the results of this study suggest that research is needed to examine how these interventions might apply to preschools with autistic children.
